# P01.36. Assessment of commercial formulations of mucuna pruriens seeds for Levodopa (L-DOPA) content

**DOI:** 10.1186/1472-6882-12-S1-P36

**Published:** 2012-06-12

**Authors:** A Soumyanath, T Denne, A Peterson, L Shinto

**Affiliations:** 1 Oregon Health & Science University, Portland, USA

## Purpose

Mucuna pruriens (mucuna) seeds contain 3-6% L-DOPA, and have been used in traditional Ayurvedic medicine to treat diseases resembling Parkinson’s disease (PD). Pilot studies in PD show that mucuna seed powder has similar effects to conventional levodopa/carbidopa medication. Formulations of mucuna seed are readily available through the internet, and are used by some PD patients as an alternative to conventional levadopa/carbidopa medication. The purpose of this study was to examine the L-DOPA content of a range of popular mucuna products in order to assess the veracity of label claims.

## Methods

Six different brands of mucuna product were ordered through the internet. Certificates of analysis were obtained where possible. A standard amount of each product was extracted using methanol: formic acid for analysis using reversed-phase high performance liquid chromatography (HPLC) with ultraviolet and fluorescence detection. L-DOPA content was calculated using a standard curve prepared using L-DOPA (Sigma-Aldrich) as reference.

## Results

The claimed L-DOPA content ranged from 25 to 250mg per dose for the six products. HPLC analysis revealed that only two of the products had L-DOPA values close to the value claimed. The remaining products contained considerably less L-DOPA , <10% in two cases, than implied on the label. Certificates of analysis suggested that not all manufacturers routinely measure L-DOPA content of their mucuna product.

## Conclusion

Four of six products examined showed a large discrepancy between label claim and L-DOPA content, independently measured by HPLC. This finding warrants further investigation as these deficiencies could impact both patients, and the outcome of clinical studies using these products.

